# Decoding properties of tRNA leave a detectable signal in codon usage bias

**DOI:** 10.1093/bioinformatics/bts403

**Published:** 2012-09-03

**Authors:** Alexander C. Roth

**Affiliations:** Swiss Institute of Bioinformatics, and Department of Computer Science, ETH Zurich, 8092 Zurich, Switzerland

## Abstract

**Motivation:** The standard genetic code translates 61 codons into 20 amino acids using fewer than 61 transfer RNAs (tRNAs). This is possible because of the tRNA's ability to ‘wobble’ at the third base to decode more than one codon. Although the anticodon–codon mapping of tRNA to mRNA is a prerequisite for certain codon usage indices and can contribute to the understanding of the evolution of alternative genetic codes, it is usually not determined experimentally because such assays are prohibitively expensive and elaborate. Instead, the codon reading is approximated from theoretical inferences of nucleotide binding, the wobble rules. Unfortunately, these rules fail to capture all of the nuances of codon reading. This study addresses the codon reading properties of tRNAs and their evolutionary impact on codon usage bias.

**Results:** Using three different computational methods, the signal of tRNA decoding in codon usage bias is identified. The predictions by the methods generally agree with each other and compare well with experimental evidence of codon reading. This analysis suggests a revised codon reading for cytosolic tRNA in the yeast genome (*Saccharomyces cerevisiae*) that is more accurate than the common assignment by wobble rules. The results confirm the earlier observation that the wobble rules are not sufficient for a complete description of codon reading, because they depend on genome-specific factors. The computational methods presented here are applicable to any fully sequenced genome.

**Availability:** By request from the author.

**Contact:** alexander.roth@isb-sib.ch

## 1 INTRODUCTION

**Fig. 1.**
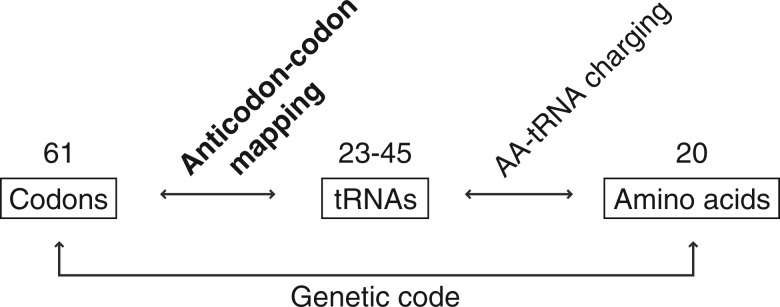
Mapping of the genetic code. In the standard genetic code, there are 61 mRNA codons that specify the 20 amino acids. The decoding carried out by 23–45 tRNA molecules, each charged with the assigned amino acid by an ARS. The mapping of tRNA anticodons to mRNA codons is the focus of this study

The standard genetic code consists of 61 sense codons and 3 stop codons, coding for 20 amino acids and a termination signal. The molecules that convey this mapping of translation are several types of transfer RNA (tRNA). To one side of the tRNA, an amino acid is attached by a designated aminoacyl tRNA synthetase (ARS). This coupling of each amino acid by ARS to the appropriate tRNA molecule is a pivotal part for the mapping of the genetic code (Ibba and Söll, 2000). Located on the other side of the tRNA is the anticodon that binds to specific codons of the messenger RNA (mRNA) at the A-site of the ribosome, ensuring that the correct amino acid is decoded. There are fewer tRNAs than codons, as non-standard base paring allows one tRNA molecule to read multiple codons, thereby reducing the number of tRNAs necessary for reading all codons. Depending on the organism, the number of different tRNA isoacceptors range from 23 (allowing for a fully degenerate binding at the third codon position) to 45 (allowing only degenerate binding of pyrimidines). This anticodon–codon mapping of the tRNAs to the mRNA is the focus of this study (see Fig. 1). We first investigate the imprint of the decoding properties of tRNA in codon usage bias, then we exploit the signal to infer the codon reading in yeast.

The codon usage bias is the non-random, unequal usage of synonymous codons. It is shaped by the balance of mutational bias and selection (Bulmer, 1991). The mutational biases originating from DNA processes (replication and repair errors, etc.) generates a variation in the sequences. These sequence variations are under various selective pressures. A prominent such pattern of selection is the co-evolution of tRNA abundance and codon usage bias (Dong *et al.*, 1996), Many of the different patterns are induced by the physiological constraints of translational selection (Plotkin and Kudla, 2011). The relative strength and importance of all factors that influence the codon usage bias has not yet been resolved. Given the central role of tRNA in translation, it is not inconceivable that a signal caused by the decoding properties of tRNA is detectable by analysis of codon usage bias.

The decoding properties of tRNA are governed by several factors. In essence, the ribosome constrains the first and the second position of the codon to strict cognate (Watson–Crick) binding, but monitors the third position of the codon less stringently (Ogle and Ramakrishnan, 2005). The third position of the codon and the first position of the anticodon are thus called the wobble position. Driven by the necessity for stable protein synthesis, the nucleotides at the wobble position of the tRNA are often chemically modified to alter the specificity of the binding (Agris *et al.,*, 2007). The cell makes a comparatively large investment into genes for tRNA modifications, to maintain tRNA stability, aid recognition for the corresponding ARS and to ensure accurate codon reading (de Crécy-Lagard, 2007).

The assignment of codon reading is important for several methods in sequence analysis. Some indices of codon usage depend on knowledge of specific anticodon–codon mapping [e.g. tAI (Friberg *et al.*, 2006) and TPI (dos Reis *et al.*, 2003)]. The mapping may help to understand the evolution of the genetic code and to find potential targets for re-engineering the genetic code to incorporate non-natural amino acids into proteins (Moura *et al.*, 2010). The most conclusive way of determining the binding characteristics (i.e. accuracy and efficacy) is via experiments (Björk and Hagervall, 2005; Johansson *et al.*, 2008). Unfortunately, experimental determination of the tRNA properties is expensive and time-consuming. Therefore, the ability to predict the decoding properties of tRNA is a highly desirable alternative to experimental assignment.

**Fig. 2.**
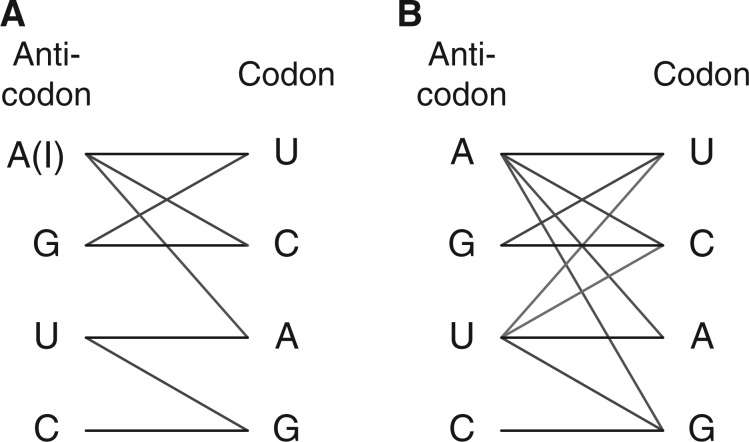
Anticodon–codon mappings. (**A**) The original wobble rules by (Crick, 1966). (**B**) Possible codon readings observed in experiments

Using knowledge of nucleotide chemistry of base pairing, Francis Crick proposed a scheme for the decoding properties of tRNA (Crick, 1966). These are the wobble rules, which still remain a common description for the anticodon–codon mapping. The original version of the wobble rules can briefly be described as: A:U, C:G, G:{C, U}, U:{A, G}, and in the case of a adenine modified to inosine: I:{A, C, U}. The latter being the only known modification when the wobble rules were devised. This notation is understood in the following way: the base at the first position of the anticodon is before the colon while the set of bases at the third position of the codon that can bind with the first position of the anticodon is after the colon. For example, U:{A, G} means that a uracil at the wobble position of the tRNA can bind either an adenosine or guanine. The original wobble rules are summarized in Figure 2A.

Over time, as new tRNA modifications were discovered, several cases were found in which the original wobble rules failed to describe the true codon reading Agris (1991); Agris *et al.,* (2007); Guthrie and Abelson (1982); Yokoyama and Nishimura (1995). In response, the original wobb rules were updated several times to accommodate the newly discovered codon readings. For example, in the restricted version of the wobble rules for eukaryote, the I:A wobble is not allowed (Guthrie and Abelson, 1982). Observed possibilities of codon reading are summarized in Figure 2B. It turns out that finding simple ‘rules’ for codon reading is not trivial and maybe impossible. To date, there is no generalization of the wobble rules, even when the base modifications are known.

A particular method extending the wobble rules worth mentioning is the wobble parsimony method (Percudani *et al.*, 1997; Percudani, 2001). This method infers the codon reading assignment in eukaryotes from genomic data. It uses the wobble rules and adds the knowledge of the presence of tRNA isoacceptors for a given organism, information that can be extracted rather easily from the complete genome (Lowe and Eddy, 1997). Wobble parsimony assumes that a codon with a cognate tRNA present, has only canonical decoding. Extended codon reading by a tRNA is assumed if a codon does not have a cognate tRNA. The rules that specify the wobble parsimony in eukaryotes are (i) codons with cognate tRNAs are assigned canonical decoding, (ii) codons without cognate tRNA are assigned following the principle of restricted wobbling (G:U, A:C) and (iii) extended pairings (A:A, U:G) are assumed for codons that remain unassigned. Wobble parsimony is only applicable for eukaryotes and not bacteria, where the tRNAs commonly recognize more codons than eukaryotes.

Another sequence-based approach to infer trends in codon reading of tRNA is based on the analysis of tRNA genes and tRNA-modifying enzymes across species (Grosjean *et al.*, 2010). Four major decoding strategies over the three kingdoms of life have been identified by the absence/presence patterns of tRNAs and genes that are involved in specific nucleotide modifications. It is assumed that the proteins for nucleotide modifications have evolved to optimize specificity and efficiency of translation. There are several exceptions for the standard modes of decoding, in particular for uracil modifications where it is difficult to distinguish the codon reading. This is a limitation of the method. For example, in a four-codon box, there is no need to prevent the U*:U reading, although it is not clear if this reading is viable or not.

In this study, we use a novel approach to detect the imprint of tRNA-decoding properties in the codon usage bias and to infer the codon reading of tRNA. We propose a Hidden Markov Model (HMM) method and two auxiliary methods: regression (REG) and codon correlation (CC). The results show that the best predictions rank high in the distribution of all solutions. Also, the three methods generally agree on the prediction. The predictions of the methods outperform the wobble rules on experimentally verified codon readings.

## 2 APPROACH

### 2.1 Prediction of anticodon–codon mapping using sequence data

The three methods use solely genomic data. In the cases of HMM and CC, the consecutive synonymous codons are used. The observation that tRNAabundance is correlated with codon usage bias is exploited in the regression method. To reduce the vast solution space of possible codon readings, we make the following assumptions:

1. We take the amino acid identity of a tRNA as given from the genomic tRNA data and assume that the aminoacylation errors by ARS are negligible, because the aminoacylation step of tRNA is much more accurate than mRNA decoding. Aminoacylation errors occur at a rate of ~ 10^−6^ (Schulman, 1991; Söll, 1990), whereas the estimated average error frequency for mRNA decoding is *<* 4×10^−4^ per codon (Parker, 1989). Also, we assume that the tRNA cross-box reading of other amino acids is negligible (Kramer and Farabaugh, 2007).
2. We make a distinction between decoding accuracy (optimizing the codon reading and avoiding errors) and decoding efficacy (optimizing translation rates). We are interested if a tRNA can read a codon and are not concerned with the speed of translation. We assume that the efficacy is orthogonal to the accuracy of reading and that rare codon readings can be detected in the codon bias. Therefore, we apply binary anticodon–codon interaction of the tRNA and mRNA binding. The assessment of the relative binding frequencies is not addressed. Codon usage can have a large influence the rate of translation (Ingolia *et al.*, 2009) and unequal reading of different codons by the same tRNA is known to influence codon bias (Curran, 1995; Ran and Higgs, 2010). On the other hand, experimental evidence in yeast shows, that all codons are translated with similar speed (Qian *et al.*, 2012).
3. We divide all possible binary mappings into two mutually exclusive subsets of codon readings. This is necessary because highly degenerated amino acids can have a large number of mappings (in the order of billions). The large number of possibilities can lead to implausible readings randomly having a higher score than the true reading. Therefore, we restrict the solution space to a set of mappings that are plausible, that is, mappings that have been observed experimentally (Fig. 2B). The other subset of non-plausible solutions is used for reference to estimate the distribution of the scores and to assess their significance. The plausible readings consider alternative solutions for the A and U nucleotides (and modifications) at the wobble position of the tRNA. The C nucleotide can only assume cognate binding (C:G) and the G nucleotide constitutively read the pyrimidine bases (G:{U, C}). This is reflected in the genetic code where all two-box pyrimidine codons have one single tRNA_NNG_. Together with the one-box codons, these cases have only one trivial solution.

## 3 METHODS

### 3.1 HMM for the tRNA states

**Fig. 3.**
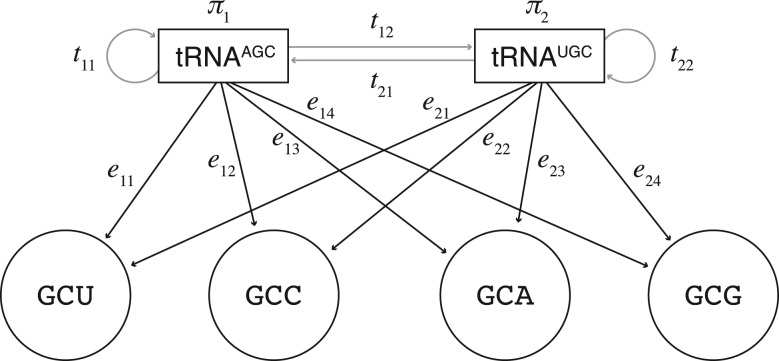
HMM for alanine in yeast. The gray arrows represent the transition probabilities *t* of changing between the unknown tRNA states. Associated with the model are the tRNA starting state probabilities *π*. The black arrows represent the emission probabilities *e* of observing the codon given the tRNA states. The transition and start probabilities are estimated from the tRNA abundances and a tRNA reuse parameter. Each model has different emission probabilities derived from the codon reading being evaluated. The model with the highest probability given the sequences is the predicted codon reading

In this study, the primary method to infer the codon reading is a discrete, finite HMM. The hidden states of the HMM are the unknown tRNAs that are used to decode the codons. The observable output of the tRNA states is the sequence of consecutive synonymous codons for one amino acid regardless of the interval size. An HMM for the codon reading of a particular amino acid is described by the initial starting probabilities *π*, the transition probabilities *t* and the emission probabilities *e* of the tRNA states. Figure 3 illustrates an example, of the structure of the HMM for alanine. For each amino acids, there can be several models, each defined by a different set of probabilities, The goal is to determine which model has the highest probability of observing the codon sequences given the parameters. From the highest scoring model, we infer the anticodon–codon mapping. The parameters of a given model are defined by the hypothetical codon reading being evaluated. No parameters of the HMM are optimized during evaluation, instead all parameters are predetermined as follows.

The matrix of emission probabilities *E* contains zero or non-zero elements. The rows represent the tRNAs for a given amino acid, and the columns represent the codons that code for that amino acid. For example, of alanine in yeast (two tRNAs and four codons), the matrix has two rows and four columns. A matrix element is positive if the tRNA can be used to decode that codon or zero otherwise. For the matrix to express probabilities, the rows of the matrix are normalized to sum to one (∑*_j_e_ij_* = 1), and the probability is equally distributed over all allowable codons. For example, if the first tRNA reads the two first codons out of four, the probabilities in the first row of *E* will be [0.5, 0.5, 0, 0].

The transition matrix *T* is a square matrix of probabilities changing between the tRNAstates, where the sum of all transitions leaving or staying in a state is one (∑*_j_t_ij_* = 1). The transition probabilities are predetermined from the tRNA frequency vector, such that probability of staying in, or moving to, a state is proportional to the tRNA abundance. We assume that the underlying tRNA dynamics is the same for the different models of codon reading and *T* is constant for all models. In agreement with observations, intra-specific codon usage bias (Cannarozzi *et al.*, 2010; Tuller *et al.*, 2010), the probability of staying in the same state is higher. We capture this by adding a linear term to the diagonal, which puts more weight on the reuse of rare tRNA. The reuse term is pre-estimated from data, with the intercept *α* = 0.065, and the gradient *β* = –0.087, by an iterative procedure that can be described as follows. Starting with an initial guess of transition probabilities (bases on tRNA frequencies), the Viterbi algorithm computes the most likely sequence of states, given the observed sequences of codons. From the inferred states, we count the number of transitions and the parameters are updated accordingly, and the procedure is repeated until the transition probabilities converge. By this procedure, we found that reuse appears to be more pronounced for tRNA coding for rare codons and therefore the weight is linearly increased for these. The values of the transition probabilities are evaluated by introducing normally distributed noise and verifying the degree of reproducibility of the results.

The initial state vector **π** = [*π*_1_*π*_2_]^T^ is computed from the eigen decomposition of the transition matrix, which gives the limiting tRNA usage. Decomposition of the transition matrix is chosen over the alternative of using the codon, or the tRNA frequency vector, because we assume that translation is an ongoing process where several ribosomes are engaged in translating the same mRNA repeatedly (Karamyshev *et al.*, 2004). Hypothetically, the tRNA concentration profile around the ribosome is different from the global average tRNA content. However, it turns out choosing either of the two alternatives have little impact of the final result.

Now that we have described how to determine the probabilities and we want to find the model that has the highest probability of describing the observed sequence of consecutive codons. All models have identical starting and transition probabilities, but the emission probabilities are different. To evaluate the match of a model *λ*= {*E, T,* **π**} and an observed sequence *O*, we need to find the probability of the codon sequence given the model *P*(*O*|*λ*). This involves considering all possible paths through the HMM, which can be solved efficiently by the forward procedure (Rabiner, 1989).

The measure that is used to compare the models for different codon reading possibilities is the combined probabilities of the HMM for all observed sequences.

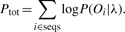

The model that gives the highest total probability is the inferred codon reading. It should be noted that we use the likelihood *P*(*O*|*λ*), rather than the posterior probability *P*(*λ*|*O*). Because we consider all models to be equally likely (i.e. the prior probability *P*(*M*) is the same for all models), the likelihood and the posterior are effectively equal. Likewise, the probability of the evidence *P*(*O*) is equal for all evaluations.

### 3.2 Regression of tRNA abundance and codon usage

The regression method (REG) exploits the systematic, positive correlation of codon usage, and tRNA abundance (Garel, 1974; Ikemura, 1981). That is, tRNA abundance in the cell is correlated with the codon usage bias, such that the most abundant tRNAs correspond to the most frequent cognate codons. For our purpose, the tRNA gene copy number is used as a proxy for the tRNA abundance (Dong *et al.*, 1996; Ikemura, 1981).

Experiments show that the tRNA abundance varies with the square of the codon frequencies (Dong *et al.*, 1996). There are theories that propose other dependencies (Ehrenberg and Kurland, 1984; Liljenström *et al.*, 1985). However, we find that the quadratic dependence fits better with known codon readings and hence this dependence is used in the model.

**Fig. 4.**
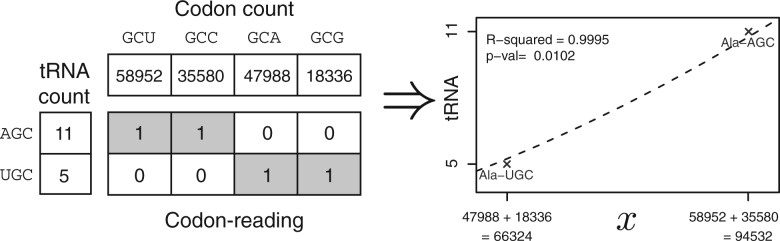
Description of the regression method. The regression method described for alanine in yeast. The reading matrix maps the tRNA gene copy number to the codons. In this example, tRNA_AGC_^Ala^ has 11 copies in the yeast genome and can read the codons {GCU, GCC}, which have frequency of 58 952 and 35 580 respectively, and transforms to 94 532 (based on the codon reading in the example). The summing can conveniently be performed by multiplying the codon vector by the reading matrix. This gives the point (94 532, 11) and similarly for tRNA_AGC_^Ala^ that read {GCA, GCG} has the point (66 324, 5). The predicted codon reading is the matrix that gives the best linear regression fit of a quadratic term of the codon usage (tRNA ~ *γx*^2^ +*e*). The regression is intercepted through the origin and the shape parameter of the quadratic term is estimated from the regression. The fit of a particular codon reading is measured by the coefficient of determination *R*^2^

The regression method predicts codon reading by evaluating which of all plausible solutions give the best fit for the relationship between codon frequency and tRNA abundance. An example of evaluating a codon reading for alanine in yeast is illustrated in Figure 4 (the fit of other amino acids are shown in Supplementary Fig. S1). For each potential binary codon reading, the codon frequency is computed as the sum of all occurrences of the codons that can be read by a given tRNA. The sum of codon counts *x_i_* that is read by the *i*th tRNA is computed by

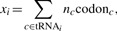

where *n_c_* is a normalization factor to account for multiple codon reading by tRNA*_i_*. The tRNA is allocated a fraction of codon counts based on the total number of different synonymous codons the tRNA can read so that each tRNA pick up the correct proportion of codon counts. The normalization factor is computed by

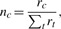

where *r* is the fraction of different codons that the tRNA can read. For example, codon *c* is read by tRNA*_i_* that can read four codons. Codon *c* can also be read by another tRNA that reads three codons. Consequently, the normalization factor then becomes *n_c_* = (1*/*4)*/*(1*/*4+13)= 3*/*7.

Now, we have the summed codon counts *x_i_* for a specific codon reading and the corresponding tRNA counts tRNA*_i_* as given from the genomic data. According to the assumed dependence, the fit of a codon reading matrix is computed by the regression model

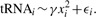


The model uses only the quadratic term and the intercept always passes through the origin. The value of the shape parameter *γ*is estimated from the data during the regression. Thus, there is only one degree of freedom and it is possible to fit two points, as in the case for amino acids with two tRNA isoacceptors. The error term *∈_i_* represents the deviations of the observed values from the expected value (i.e. the residual variance that is not explained by the model). Here, we have chosen to evaluate the fit of the model by the coefficient of determination *R*^2^.

### 3.3 Autocorrelation of synonymous codons

There is a strong preference for using an identical codon at the next consecutive instance of an amino acid. This stems from several prominent biases, such as a codon bias toward optimal codon and variable GC content over the genome. As a result of these evolutionary forces, there is positive autocorrelation of these codons. Here, we exploit this phenomena for analyzing codon reading. This method is from now on referred to as CC, not to be confused with the correlation of codon usage and tRNA abundance.

**Fig. 5.**
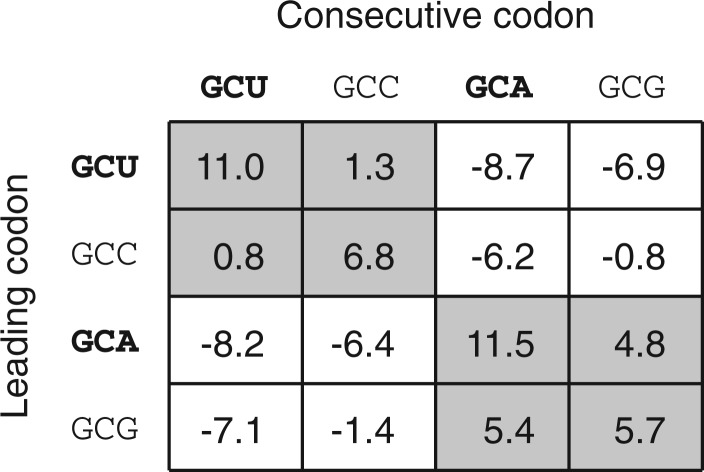
Codon correlation of consecutive synonymous codons. Matrix of the correlation of consecutive codons for alanine in yeast. The numbers are the standardized normal transformed deviations from the mean (*Z*-scores). For example, the observed count of GCU followed by another GCU is 11.0 standard deviations more frequent than expected. Two blocks of positive Z-scores (shaded) coincide with the codon reading of the two tRNAs. This observation is exploited to infer codon reading

Figure 5 shows a matrix of codon correlation for alanine in yeast (other amino acids are shown in Supplementary Fig. S2). This matrix is constructed from the counts of consecutive synonymous codons at the instances of the amino acid regardless of the distance between them for all sequences meeting the requirements (see below). The counts are measured in *z*-scores, that is, they are compared with expected usage of codon occurrences. This is done by subtracting the expected value from the observed counts and dividing by the standard deviation, *Z* = (*X*–*E*[*X*])*/σ_X_*, assuming that the codon counts are independent events and follow a binomial distribution. The *z*-transformed matrix give the observations in standard deviations away from the mean. As expected, the diagonal has large positive values as expected from prevalent biases (e.g. codon usage bias, nucleotide distribution, etc.). More interesting are the positive entries at the off-diagonals. These entries often coincide with known codon reading of isoacceptor tRNA and codons read by the same tRNA appear as block structures in the matrix (Fig. 5). These block structures are exploited to predict the codon reading.

A potential codon reading is evaluated as follows. The sum *s* of the counts of codon pairs read by the same tRNA is computed by summing the elements of the matrix of observed counts *X*, according to the codon reading being evaluated. If an element of the matrix is read by the same tRNA, the count is added, if not, it is subtracted. The diagonal that represents the bias for reusing the same codon at consecutive instances is ignored. This procedure is described by

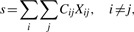

where *C_ij_* is +1 if the codons are read by the same tRNA and –1 if not.

The same procedure is performed for the matrix of ‘expected’ counts of consecutive codons. Thereafter, the *z*-transformation gives the total score by subtracting the total expected count from the total observed count and dividing by the total standard deviation. This provides the procedure to evaluate different reading matrices. The codon reading matrix that gives the highest total *z*-score is the predicted anticodon–codon mapping.

### 3.4 Sources and preparation of data

The comparisons in this study all use the same sources of sequence data. The data for the tRNA gene copy number come from the ‘Genomic tRNA database’ (Chan and Lowe, 2009). Codon usage frequencies are taken from published genome sequences (Kersey *et al.*, 2010). In the process of assembling codon usage frequencies, the sequence data are filtered to remove sequences that have non-conforming codons, such as: internal stop codons, programmed frame-shifts and undetermined nucleotides. Sequences shorter than 50 amino acids are discarded. For the synonymous codon pair counts of an amino acid, sequences with less than three instances of that amino acid are excluded. Other experimental reference data compiled from various sources are mentioned in the respective parts of the sections below.

## 4 RESULTS AND DISCUSSION

In this section, we will validate the methods and benchmark them, first by checking where the most plausible codon readings are in the solution space. Then, we use a consensus procedure to see how often the three different methods agree with each other. Finally, we compare the predictions against experimental evidence. Using these methods and experimental evidence, we suggest an improved prediction for codon reading in yeast.

### 4.1 Plausible codon readings rank high in the solution space

**Fig. 6.**
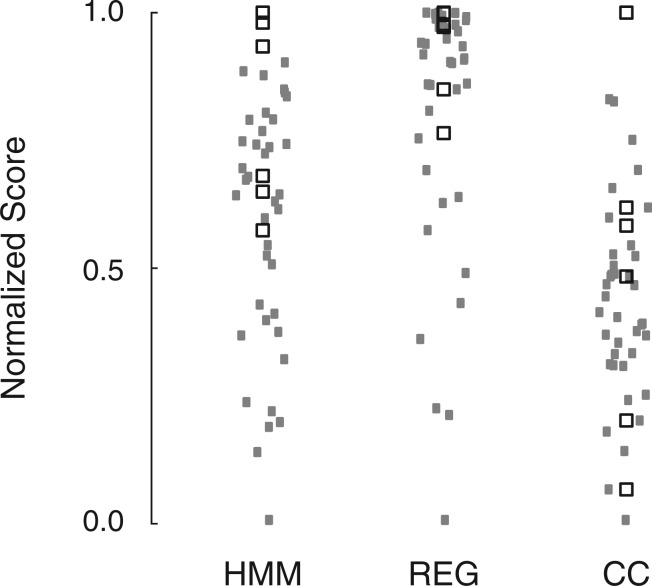
The relative distributions of the scores of the three methods for alanine in yeast. Each point represents the relative score on the *y*-axis of all the possible binary codon readings. The scores are scaled to range from 0 to 1. The values have randomness added to the *x*-coordinate to better visualize the distribution. The subsets of codon readings that are plausible, are drawn as black squares. These codon readings rank high among the other solutions, drawn as gray points

To investigate the evolutionary signal in the codon usage bias due to the physiological constraints of tRNA decoding, we look at how the subset of plausible codon readings rank in comparison with other binary codon readings. The plausible codon readings are based on arguments of nucleotide chemistry and on trends in the observed anticodon–codon mappings (the possibilities are depicted in Fig. 2B). The distributions of the scores of the three methods for alanine in yeast are illustrated in Figure 6, where the subsets of plausible codon readings are indicated by black squares and the other implausible solutions by gray points. The scores are normalized to range from 0 (lowest) to 1 (highest) by *s*_n_ = (*s*-min*s*)*/*(max*s*-min*s*). The best plausible solution for alanine ranks in the top of the distribution for all three methods. For REG and HMM, all plausible solutions are in the upper half of the distribution, whereas in the CC methods have a larger spread.

Repeating this for all non-trivial amino acids in yeast shows that the highest scoring plausible codon readings tend to be at the top of the distributions (see Fig. S3). For CC, REG and HMM, all the plausible solutions are in 25, 58 and 75% of the upper half part of the distribution, respectively. The method of codon correlation has the largest variation and the subset is often spread through the rest of the distribution. Of the three methods, the HMM has plausible solutions that generally rank high in the distribution.

Next, we compare how the predictions compare against random. Randomizing the codon usage using the global codon usage of the organism removes the signal of codon reading. The randomization for CC and HMM is produced by taking the original amino acid sequences and drawing random synonymous codons according to the codon frequencies. The REG method is randomized by drawing from a skewed normal distribution based on the codon usage of the genome. The best plausible solution is compared with the best plausible solutions of randomized data.

To compute the significance of our solutions, we compare 99 randomized sample points with the real solution. We use empirical *P*-values based on a Monte Carlo procedure, which is based on rank and do not rely on assumptions of the distribution. The *P*-values are calculated by 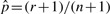, where *n* is the total number of simulations and *r* is the number of simulations that are equal or greater to the actual, non-randomized data value. Table 1 lists the empirical *P*-values of the solutions for amino acids in yeast. The solutions for the HMM method are all significant with *P*-values from 0.01 to 0.02, which express that the solutions of HMM ranks first or second in comparison with the simulated data. This is not the case for CC and REG, which produce only a few solutions that are significantly different from random assignment. In particular for the atypical codon reading of arginine, the CC method performs poorly where the majority of the simulated solutions have better scores then the actual data. Although the block structures of the CC method often agree with known cases, CC tend to have less predictive power than REG and HMM, likely from the inherent limitations of the CC method to predict cases broad codon readings where the tRNAs can read multiple codons. In summary, these observations are indications of the signal of tRNA decoding properties in coding sequences.

**Table 1.** Results of randomization for non-trivial amino acids in yeast

Amino acid	CC	REG	HMM
Alanine	0.01	0.10	0.01
Arginine	0.97	0.48	0.01
Glycine	0.20	0.31	0.01
Leucine	0.12	0.31	0.02
Proline	0.40	0.15	0.02
Serine	0.25	0.22	0.01
Threonine	0.06	0.06	0.01
Valine	0.04	0.07	0.02

The best solution for each method is compared against solutions using randomized codon usage. The HMM method compares favorably, because all the solutions are significant (*P*-values: 1–2%).

### 4.2 The three different methods often agree on the result

**Fig. 7.**
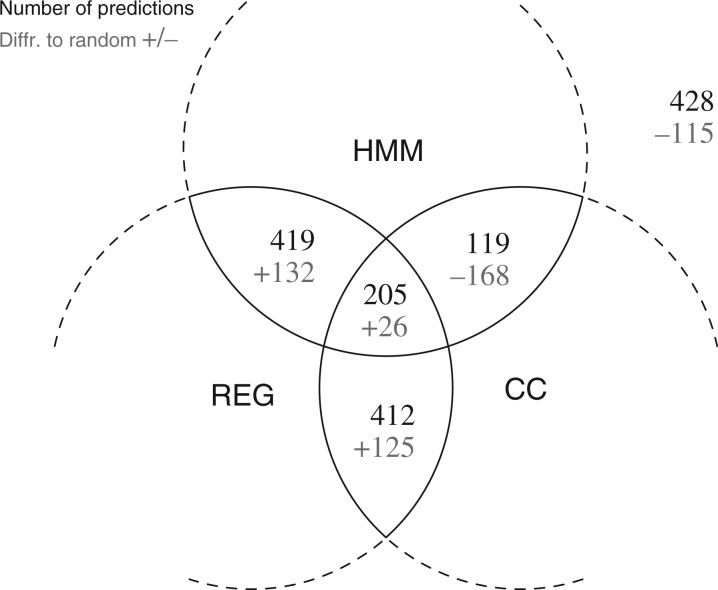
Consensus among the sequence-based methods. The black numbers show the number of predictions that coincide from the different methods. The gray number below the black numbers indicate the difference to random. For example, in the middle intersection there are 205 predictions where all three methods agree, which is 26 more than what is expected by random. All values are significantly different from random

Agreement among the predictions by the methods may give an indication of the ability of the methods to detect the signal. To examine this, we count how the predictions of each method coincide for the codon reading of non-trivial amino acids with codon degeneracy larger than three in 241 genomes. This yields a total of 1583 predictions. Each of the predicted solutions is compared with the distribution of the best plausible solution from several randomized sequence data (generated as described above). The result of the comparison is summarized as a Venn diagram in Figure 7. In 205 cases, all three predictions agree, which is 26 cases more than the expected value of 179. This result is statistically significant with the *P*-value = 0.012, computed using the Monte Carlo procedure as described above. From the other perspective, the instance where all three methods disagree has a count of 428, which is 115 less than expected (*P*-value *<* 10^−10^). For 73% of the 1583 predictions, at least two methods are in agreement. The HMM and REG methods have the highest number of agreements, 419 times, which is slightly more than REG and CC (412). HMM and CC have only 119 predictions that agree, which is 168 less than expected by chance. This is somewhat unexpected, because HMM and CC both use codon pair frequencies for their predictions. This discrepancy is possible due to the inherent shortcoming for CC to capture broad codon readings. In summary, the observation that the methods agree with each other more often than expected by random supports the notion that codon reading can be detected from codon usage bias.

### 4.3 Validation shows significant agreement with experimental data

To further evaluate the performance of the methods, we compare the predictions with experimentally determined codon readings. This is to establish which of the methods perform best and to compare sequence-based methods with the simplistic method of wobble parsimony that is commonly used for yeast. The experimentally verified codon readings and the predictions are listed in Table 2. The wobble parsimony rules describe the codon reading correctly in 2 out of 11 cases (18%) in yeast. A result that is worse than a random assignment, pointing out the limitations of specialized rules. In fact, generalizing codon readings is not viable even where the tRNA modifications are known, because the influence of base modifications depends on the context. For example, tRNA_ncm^5^UGU_^Pro^ can read the entire codon box, whereas the same modification in serine tRNA_ncm^5^UGA_^Ser^ only reads the purine ending codons {TCA TCG}. Hence, *four-box* codon families can have the same base modifications at the wobble position, with different codon reading. Wobble parsimony is only applicable to eukaryotes, and if applied to bacteria in Table 2, not a single case would be correct.

**Table 2.** Evaluation of methods

	Anticodon	Org	Read	WP	CC	REG	HMM
Arg	mcm^5^UCU*^a^*	*S.c*	R				✓
Gln	mcm^5^s^2^UUG*^a^*	*S.c*	R			✓	✓
Glu	mcm^5^s^2^UUC*^a^*	*S.c*	A	✓	✓	✓	✓
Gly	mcm^5^UCC*^a^*	*S.c*	R		✓		
Leu	UAG*^a^*	*S.c*	N		✓		
Pro	ncm^5^UGG*^a^*	*S.c*	N			✓	✓
Ser	IGA*^a^*	*S.c*	D			✓	✓
Ser	ncm^5^UGA*^a^*	*S.c*	R			✓	
Thr	ncm^5^UGU*^a^*	*S.c*	R		✓		
Val	IAC*^a^*	*S.c*	Y	✓	✓	✓	
Val	ncm^5^UAC*^a^*	*S.c*	R		✓		
Ala	cmo^5^UGC*^e^*	*S.e*	N	NA			✓
Thr	cmo^5^UGU*^e^*	*S.e*	V	NA	✓		
Val	cmo^5^UAC*^e^*	*S.e*	N	NA			✓
Leu	cmnm^5^UAA*^d^*	*E.c*	R	NA	✓		✓
Leu	cmo^5^UAG*^c^*	*E.c*	D	NA			
Val	cmo^5^UAC*^b^*	*E.c*	N	NA			
Val	mnm^5^UUU*^f^*	*T.t*	R	NA		✓	✓

	Total correct assignments:			2	8	7	9

Four methods are evaluated against experimental evidence. The methods are WP = wobble parsimony, CC = codon correlation, REG = regression and HMM = hidden Markov model. The parentheses indicate that the codon is read by the tRNA when the tRNA is over-expressed, which here is considered to be a true codon reading. The experimental data originate from the following publications: (Johansson *et al.*, 2008)*^a^*, (Mitra *et al.*, 1977)*^b^*, (Sørensen *et al.*, 2005)*^c^*, (Takai *et al.*, 1994)*^d^*, (Näsvall *et al.*, 2007)*^e^* and (Murphy *et al.*, 2004)*^f^*. The organisms (Org) are: *S.c*: *Saccharomyces cerevisiae*, *S.e*: *Salmonella enterica*, *E.c*: *E.coli* and *T.t*: *Thermus thermophilus*. The experimentally determined codon reading of the tRNA (Read) are indicated by standard IUPAC names (e.g. R={A, G}, D={U, A, G}, etc.). Agreement of a prediction with experimental evidence is indicated by a checkmark (✓). The total number of correct predictions for the different methods are given in the last row. The HMM method produces the best results and is the only method that significantly agree with the experimentally determined codon readings. Wobble parsimony performs poorly for yeast (*S.cerevisiae*) and is not available for bacterial genomes (NA).

Of the sequence-based methods, the HMM performs best, where nine (of 18) of the predicted codon assignments are correctly assigned, followed by CC (8 of 18) and REG (7 of 18). A random assignment will be correct on the average five times and HMM is the only method that is significantly different from a random assignment (*P*-value = 0.04), based on a binomial distribution of the subset of plausible solutions. Note that with only 18 data points, it is difficult to get significant results, because the lack of experimental data limits the power of the test and that the data may itself contain errors.

### 4.4 A better description of the codon reading in yeast

Here, we summarize what is known about codon reading for cytosolic tRNA in the yeast *S.cerevisiae* based on experimental results and the predictions from the sequence-based methods. The HMM method performs the best and is therefore used for the bulk of the predictions. When there are ambiguities in the predictions, we rely partly on manual curation. For example, when CC an REG both agree on a solution that coincides with the second best HMM prediction, we choose this prediction. Regardless of the predictions, we trust the experimental data and these assignments take precedence (Johansson *et al.*, 2008).

**Fig. 8.**
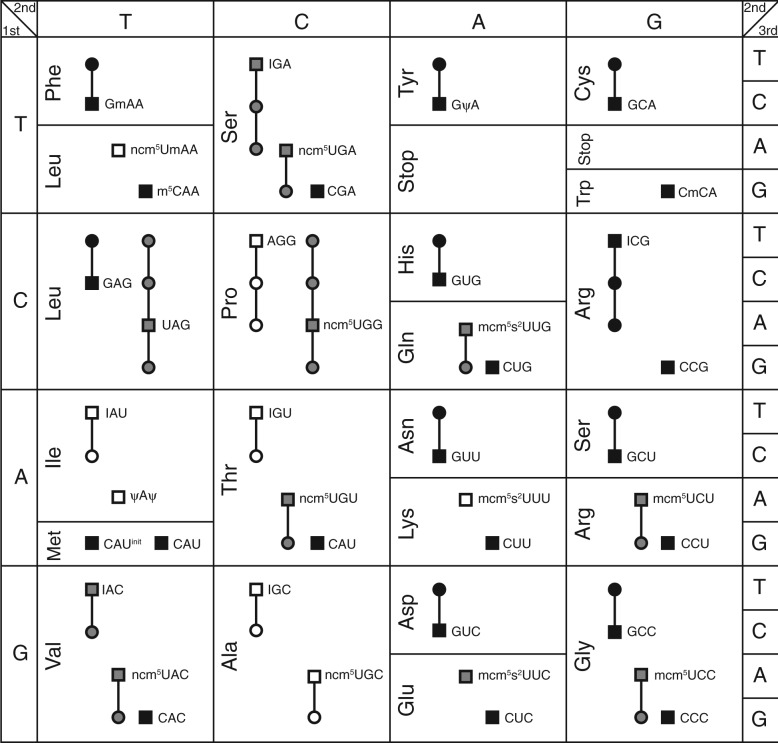
The genetic code and decoding abilities of individual tRNA species in yeast. Symbols connected with a line indicate that the codons are read by the same tRNA. Square symbols indicate codons with a cognate tRNA and circles are codons without. The symbols in gray color are the codon readings that are experimentally determined and those in white are computationally predicted from sequence data. Black symbols indicate cases where the codon reading is trivial, that is, there is only one tRNA to decode the codons

The codon reading assignment for all tRNAs in yeast is depicted in Figure 8, overlaid onto the genetic code (see also Supplementary Table S1). In summary, in yeast there are 42 different tRNA isoacceptors, of which 23 tRNAs have trivial solutions, 11 have previously been experimentally determined (Johansson *et al.*, 2008) and 8 are predicted by the methods herein. The predictions have substantial differences compared to the prediction using the wobble parsimony method, where 12 of the 19 non-trivial tRNAs have different assignments than ours. The common feature of the discrepancy is that the base modification at the wobble position allow broader specificities to promote a functionally redundant decoding system than the codon readings predicted by wobble parsimony.

Inspection of the codon reading in yeast shows cases that depart from the common pattern in eukaryotes. For the amino acids, alanine, proline and leucine, the tRNA with C at the wobble position is absent. The NNG codons can therefore only be decoded via wobble reading. Another deviation from the anticipated decoding strategy is arginine, which lacks the common tRNA to decode codon CGA. This implies that this codon is read by the tRNA_ICG_^Arg^ (adenine modified to a inosine at the wobble position). The CGA codon is not rare in yeast, suggesting that the I:A reading is not inefficient, contrary to *Escherichia coli* (Curran, 1995). This decoding strategy for arginine is seen in several other fungi. Glycine is another special case for eukaryotes in that it does not use tRNA_ACC_^Gly^ (or any of the derivatives), although the cognate GGT is often a frequent codon. Furthermore, tRNA_mcm^5^UCC_^Gly^ is a modification that in yeast is otherwise only observed for purine 2-codon family boxes, which hints toward special properties for this codon box. The tRNA_UAG_^Leu^ in yeast is uncommon for eukaryotes in that it has an unmodified U at the wobble position, allowing the uracil to read all four nucleotides. The decoding of the 2-codon family boxes follows the expected pattern of one tRNA to decode pyrimidines and two tRNA for purines, although the codon reading for purines differs among amino acids. Finally, the tRNA that reads the isoleucine ATA codon contains the modification to pseudouridine tRNA*_ψ_*_A_*_ψ_*^Ile^, preventing cross reading of the AUG codon coding for methionine.

### 4.5 Sequence-based methods for codon reading prediction

In this section, we discuss the two auxiliary methods (CC and REG) and the main sequenced-based method (HMM).

#### 4.5.1 CC

A striking example of the tRNA imprint in the codon usage bias is the occurrence of block structures in the *z*-transformed tables of codon pairs for the CC method (Supplementary Fig. 5 and Supplementary S2). Several factors are likely to contribute to the off-diagonal cross correlation of codons. There is a strong variation in codon frequencies between low and high expression genes, where highly expressed genes have a codon usage that is biased toward codons corresponding to frequent tRNAs. Consequently, the next codon is more likely to be more frequent than average. In fact, there is a positive correlation between synonymous codons on the same gene, whether or not they are successive. There is a higher abundance of rare codons at the beginning of the gene for slowly loading the ribosomes, to avoid translational congestion (Tuller *et al.*, 2010). Also related to the dynamics of translation is the preferential reuse of the tRNAs (Cannarozzi *et al.*, 2010). The relative concentrations of different tRNAs depend on growth conditions of the cell (Dong *et al.*, 1996). Genes expressed under different conditions might have different codon usages and different tRNA pools that they are adapted to (Elf *et al.*, 2003). Together, the codon usage is shaped by these tRNA-related effects and their decoding properties. There are two cases where the CC method is not able to handle. One is in the hypothetical case where all tRNAs can read all codons for an amino acid. The other is that the method is of limited use for amino acids that are decoded by two codons.

#### 4.5.2 REG

The regression method is an intuitively appealing method based on the correlation of tRNA abundance and codon usage bias (Ikemura, 1981). However, there are some potential shortcomings of the REG method. Transfer RNAis not constitutively expressed and the abundance levels may fluctuate depending on regulation, which is ignored. Also, some organisms that have low-tRNA copy numbers, which decreases the resolution of the prediction. Like for all methods, the binary matrix that have the highest score constitute the predicted codon reading for an amino acid. Thus, the data determine positive cases of codon readings. For example, a codon reading that is experimentally observed only during tRNA over-expression may still leaves a trace in the codon bias. Consequently, it is considered to be viable codon reading.

#### 4.5.3 HMM

The HMM is a versatile approach that is based on establish statistics (Rabiner, 1989). As for CC, the HMM is based on the assumption that there is a signal in the successive pairs of synonymous codons. Although the HMM is appealingly mimicking the physical process of elongation by suggesting reuse of tRNA, this is no requirement. The main problem with the HMM is the large solution space for the parameters defining the model. The amount of possible solutions is reduced to a computationally, feasible size by making crucial assumptions (as mentioned above). The predetermined values of the transition probabilities are evaluated by introducing random noise. It turns out that the model is dominated by the emission probabilities, rater than the transition and the start probabilities, which can tolerate mis-specifications to at least 10%. Although, in ambiguous cases, where the first and the second plausible solutions are very close, the robustness of the parameters are lower. These are cases that require additional evidence and manual curation.

Finally, which applies to all methods, a natural question is to ask how the analysis changes using only highly expressed genes. It is well established that the degree of codon usage bias increases with expression level. Therefore, the signal might be stronger using sequences with the expression levels estimated from protein abundance data. It turns out that it does not improve on the results. A possible reason for this it that in the subset of highly expressed genes other effect than decoding dominates the selection on codon bias. Also, these sequences contain the few instances of rare codons, which weakens the signal of rare codon readings. This highlights the multiple underlying causes of codon bias and that the strength of selection is different among genes. Including as much information as possible in the analysis strengthen the signal of codon reading.

## 5 CONCLUSION

In this study, we investigate the mapping of anticodons to codons. We examine the weak, yet detectable, imprint in codon bias of selection from tRNA-decoding properties. We show that we can detect a signal that coincides with known codon reading of tRNAs. For this, we are using three sequence-based methods: an HMM, regression and CC. The predicted codon–anticodon mappings often agree with experimental results, in particular for the HMM method, which is better than the other two methods and outperforms the method of wobble parsimony. Furthermore, all three different methods significantly agree on the predictions and the predictions generally rank high in the solution space.

Associated with the methods are crucial assumptions made to aid the prediction, because the solution space is enormous. As we show, the results based on these assumptions are sufficient to make predictions that agree with experimental results. This provides a valuable basis for further studies which can improve both the model and the assumptions.

We proceed to exploit the detectable patterns of tRNA decoding to predict codon reading in yeast. We use experimentally determined codon readings, sequence-based predictions and manual curation. These novel assignments are more accurate than the wobble parsimony mappings that are conventionally used. We advise against solely using the wobble parsimony, because organisms tend to use a functionally redundant broad recognition of codons. Cases where the predicted anticodon–codon mapping deviates from common decoding patterns are interesting targets for further experiments and theoretical investigations. With the HMM, we present a statistically sound method for identifying these targets.

*Conflict of Interest*: none declared.
